# Prevalence of disturbed eating behavior and associated symptoms of anxiety and depression among adult males and females with type 1 diabetes

**DOI:** 10.1186/s40337-018-0209-z

**Published:** 2018-09-11

**Authors:** Line Wisting, Torild Skrivarhaug, Knut Dahl-Jørgensen, Øyvind Rø

**Affiliations:** 1 Regional Department for Eating Disorders, Division of Mental Health and Addiction, Oslo University Hospital, P.O. Box 4956 Nydalen, N-0424 Oslo, Norway; 2 Oslo Diabetes Research Centre, P.O. Box 4956 Nydalen, N-0424 Oslo, Norway; 3 Department of Paediatric and Adolescent Medicine, Oslo University Hospital, P.O. Box 4956 Nydalen, N-0424 Oslo, Norway; 4 Institute of Clinical Medicine, Faculty of Medicine, University of Oslo, Problemveien 7, N-0315 Oslo, Norway; 5 The Norwegian Diabetic Centre, Sponhoggveien 19, N-0284 Oslo, Norway; 6 Institute of Clinical Medicine, Mental Health and Addiction, University of Oslo, Problemveien 7, N-0315 Oslo, Norway

## Abstract

**Background:**

The increased prevalence of disturbed eating behaviors (DEB), depression, and anxiety in type 1 diabetes (T1D) is generally well established; however the majority of existing research to date has focused on female adolescents and young adults. Data on males and older females is scarce. The aim of this study was to assess prevalence of DEB and symptoms of depression and anxiety among adult males and females with type 1 diabetes, to investigate differences between individuals scoring below and above the cut-off on psychopathology, and to examine patterns of eating disorder psychopathology by age and weight.

**Methods:**

A total of 282 adults with type 1 diabetes aged 18–79 years participated in the study. Measures included the Diabetes Eating Problem Survey – Revised (DEPS-R), the Hospital Anxiety and Depression Scale (HADS), and clinical data from the Norwegian Quality Improvement of Laboratory Examinations (NOKLUS) system.

**Results:**

A total of 20.3% of the whole sample (13.3% among males and 24.8% among females) scored above the DEPS-R cut-off score for DEB. As for depression and anxiety, the prevalence in the whole sample was 6.2% and 19.0%, respectively. The prevalence was generally higher in females than males across all psychopathology measures. HbA1c was significantly associated with the DEPS-R total score (*p* < .01) among females, but not with depression and anxiety. Mean DEPS-R score decreased with increasing age, and when our previous reported data from children and adolescents are included, a peak prevalence in DEB in adolescence and young adult age is demonstrated.

**Conclusions:**

The results of this study point to the need for increased awareness of psychological comorbidity among adults with type 1 diabetes, in particular young adult females. Screening is recommended to secure early detection and subsequent intervention for these individuals.

## Plain English summary

Although increased rates of disturbed eating behaviors (DEB), depression, and anxiety in type 1 diabetes (T1D) are generally reported, data on males and older females is lacking. This study examined frequency of DEB, depression, and anxiety among 282 adult males and females with T1D across a wide age span. Approximately one-fifth of the participants (and a quarter of all females) scored above cut-off for DEB, 6.2% scored above cut-off for depression, and 19.0% for anxiety. The prevalence was generally higher in females than males across all psychopathology. HbA1c (a measure of long term blood glucose levels) was significantly associated with DEB, but not with depression and anxiety. Symptoms of DEB decreased with increasing age, and when our previous reported data from children and adolescents are included, a peak in levels of DEB during late adolescence and young adulthood was observed. Therefore, increased awareness of psychological comorbidity among adults with T1D is warranted, especially among young adult females.

## Background

Eating disorders are characterized by a restricted or chaotic food intake, a morbid preoccupation with food, weight, and shape and a distorted body image (1). The Diagnostic and Statistical Manual of Mental Disorders (DSM-5) defines the eating disorders anorexia nervosa, bulimia nervosa, binge eating disorder, other specified feeding and eating disorders and unspecified feeding and eating disorders [1]. Additionally, there are reports of some lower degree eating disturbances which do not meet frequency- or severity criteria for a formal eating disorder diagnosis as defined by diagnostic manuals such as the DSM-5. These disturbances have been defined as disturbed eating behaviors (DEB) [2], a term which will be used to describe all eating disorder psychopathology in this study.

Individuals with type T1D have been found to be at risk of developing DEB, with prevalence rates in T1D reported to be 2–3 times higher in individuals with T1D compared to healthy controls [3–5]. Possible contributing factors include the weight loss accompanied with T1D onset, the weight gain subsequent to diagnosis and initiation of intensive insulin therapy [6], and the required monitoring of food intake in order to plan insulin dosing. Finally, the availability of insulin omission as a unique, T1D-specific compensatory behavior, reported in up to 37% of females with T1D [7], is considered a potential risk factor for developing DEB in T1D. This comorbidity is associated with accelerated onset of T1D complications and increased rates of mortality compared with individuals with T1D without disturbed eating [7, 8].

The eating disorder literature has generally focused the most on adolescent and young adult populations, but increased attention has acknowledged the presence of eating disorder psychopathology also in older samples [9]. It is likely that this will be reflected in adult T1D samples as well. Although the increased prevalence of DEB in T1D is generally well established, a majority of existing research to date has focused on female adolescents and young adults [10, 11]. There is a lack of knowledge about levels of DEB among males and older females with T1D. It is known that risk of DEB varies with age and weight in the general population and among children and adolescents with T1D. It is unclear whether this is true for adults with T1D.

Mood and anxiety disorders have been found to be the most common psychiatric comorbidity in eating disorder populations [12]. Similarly, an array of research has documented increased prevalence of depression and anxiety in individuals with T1D compared to healthy controls [10, 13, 14]. Furthermore, although a majority of the literature reports positive correlations between psychopathology and poor metabolic control [15–19], other studies have yielded mixed support for these associations [20–23].

This study aimed to **i)** assess the prevalence of DEB and associated symptoms of depression and anxiety among adult males and females with T1D; **ii)** investigate differences between individuals scoring below and above the cut-off on psychopathology, with a specific focus on metabolic control; and **iii)** examine patterns of eating disorder psychopathology by age and weight.

## Methods

### Design

This is a cross-sectional design study.

### Procedure

Patients with T1D were recruited from the Norwegian Diabetic Centre (NDC) between February 2016 and October 2017. The NDC is an outpatient clinic for adults (approximately 1300) with T1D, located in Oslo. Patients from Oslo and surrounding areas are referred to the NDC by both general practitioners and hospitals. The NDC is a multidisciplinary clinic organized under the Norwegian Health South-East Authority. Questionnaires were completed as part of a routine T1D consultation at the outpatient clinic. The regional ethics committee approved the study, and written consent was obtained from all participants.

### Measures

The Diabetes Eating Problem Survey – Revised (DEPS-R) [24] is a diabetes-specific screening tool for disturbed eating and consists of 16 items. Responses are scored on a 6-point Likert scale and higher scores indicate greater pathology. A recommended cut-off score of > = 20 has been empirically established as a threshold indicating the need for further clinical assessment of eating pathology [24]. The DEPS-R has been translated and validated in a Norwegian adolescent sample aged 11–19 years [25].

The Hospital Anxiety and Depression Scale (HADS) [26] was developed to map symptoms of anxiety and depression in patients in treatment of somatic diseases. Somatic symptoms on anxiety and depression are therefore avoided in HADS in order to prevent that somatic illnesses would be misinterpreted as symptoms of anxiety and depression. HADS consists of two subscales, measuring anxiety (HADS-A) and depression (HADS-D), in addition to the total score. The Norwegian version of HADS has previously demonstrated satisfactory psychometric properties [27]. A cut-off score of ≥8 is often used for the two subscales to indicate symptoms of anxiety and/or depression need for further evaluation, whereas a cut-off score of ≥11 is used to indicate a case (i.e. moderate to severe symptoms). T1D is often associated with fear of hypoglycemia, fear of complications, and diabetes distress [10, 17], which may lead to elevated scores on the HADS. To minimize the risk of over pathologizing due to such diabetes-specific aspects, the current study therefore adopts the cut-off score of 11 to report prevalence of symptoms of depression and anxiety. However, as control data has been carried out using a cut-off of 8, rates based on this cut-off score will also be briefly reported for comparative purposes.

BMI was calculated based on self-reported weight and height (kg/m^2^), and further categorized into the following four groups according to the World Health Organization classification scheme [28]: underweight (BMI < 18.5), normal weight (BMI ≥ 18.5–24.9), overweight (BMI ≥ 25–29.9), and obese (BMI ≥ 30).

Age was categorized based on the Center for Disease Control and Prevention (CDC) age groupings [29]: 15–24 years (youngest person was 18 in the current study), 25–34 years, 35–44 years, 45–54 years, 55–64 years, and ≥ 65 years. These age groups are used in previous ED research [30]. Given the relatively low number of participants > 55 years, the two latter groups were collapsed, yielding a total of five age groups (18–24 years, 25–34 years, 35–44 years, 45–54 years, and 55 years and above).

Clinical data was assessed via the Norwegian Quality Improvement of Laboratory Examinations (NOKLUS) system, and was conducted as part of standard clinical T1D assessment at the Norwegian Diabetic Centre. T1D clinical data include HbA1c, T1D onset, and treatment mode. HbA1c is a measure of long term blood glucose levels and reflects average blood glucose the preceding 8–12 weeks. HbA1c is used here as a measure of metabolic control. A reasonable HbA1c target for many nonpregnant adults is < 7.0% (53 mmol/mol). The providers might reasonably suggest a more stringent HbA1c goals such as 6.5% (48 mmol/mol) for selected individual patients if this can be achieved without significant hypoglycemia or other adverse effects of treatment (i.e., polypharmacy) [31].

### Data analyses

Pearson correlations were conducted to investigate associations between variables. In line with Cohen [32], correlations of .10 to .29 were interpreted as small, .30 to .49 as medium and .50 to 1.0 as large. Independent samples t-tests were carried out to investigate group differences. Pearson chi-squares were used for categorical variables. Alpha level was set to *p* < .05. Effect sizes were calculated by means of Cohen’s *d*. Following the guidelines by Cohen [32], effect sizes > 0.2 were interpreted as small, > 0.5 as medium and > 0.8 as large. Statistical analyses were conducted using SPSS version 23 (SPSS IBM, NY, USA) [33].

## Results

### Participant characteristics

A total of 282 males and females aged 18–79 years (60% females) participated in the study (mean age 42.11; SD: 15.19). Table 1 illustrates sample characteristics. Mean age of T1D onset was 15.14 (SD: 11.18), mean HbA1c was 7.75% (SD: .91), and mean BMI was 25.96 (SD: 4.13). A total of 56.3% administered insulin with an insulin pen and 43.3% with a pump. All patients used basal/bolus insulin treatment, none fixed premixed regimens.

**Table 1:** Participant characteristics

	All *N* = 282	Males *N* = 112 (40%)	Females *N* = 170 (60%)	Sig. level	Effect size
Age	42.11 (15.19)	44.57 (15.92)	40.47 (14.49)	.05	.27
Diabetes onset (years)	15.14 (11.18)	15.43 (10.92)	14.94 (11.38)	ns	–
HbA1c (%)	7.75 (.91)	7.61 (.89)	7.85 (.91)	ns	–
Diabetes duration (years)	27.09 (14.44)	29.14 (14.82)	25.71 (14.05)	ns	–
BMI self-report	25.96 (4.13)	26.47 (3.82)	25.63 (4.30)	ns	–
Mode of insulin treatment	56.3% pen 43.3% pump	60.9% pen 38.0% pump	53.4% pen 46.6% pump		

Data are mean (SD). Significance level (*p* < .001, .01, and .05) and effect size (ES) estimation (Cohen’s *d*) is calculated when differences are significant. *Ns* not statistical significant differences

### Prevalence of disturbed eating behavior, depression, and anxiety

A total of 20.3% of the whole sample scored above the established DEPS-R cut-off score for DEB (Table 2). When split by gender, 13.3% of the males and 24.8% of the females scored above the cut-off score. As for depression and anxiety, when using the cut-off score of 11, the prevalence in the whole sample was 6.2% and 19.0%, respectively. In males only, the prevalence was 3.6% for depression and 8.1% for anxiety. The prevalence was generally higher in females than males across all psychopathology scores, with rates of depression and anxiety at 7.8 and 26.4%.

**Table 2:** Prevalence rates of DEB, anxiety, and depression, in adult males and females with T1D, based on a cut-off score for DEB of 20 or more on the DEPS-R, and 11 or more on the HADS subscales anxiety and depression

	All	Males	Females	*P*-value
DEB	20.3%	13.3%	24.8%	.05
Depression	6.2%	3.6%	7.8%	ns
Anxiety	19%	8.1%	26.4%	.001

The Chi-square test for independence was used to investigate whether the proportion of individuals scoring above the cut-off scores on the DEPS-R as well as the HAD anxiety and depression subscales, was significant different for males and females

Prevalence rates for depression and anxiety when using a cut-off score of 8 was also assessed, yielding prevalence rates of 13.8% and 35.4% for depression and anxiety for the whole sample. When split by gender, the prevalence of depression and anxiety was 11.8% and 24.3% among males and 15.1% and 42.9% among females.

The frequency of participants scoring above the cut-off score one more than one of the psychopathology measures (disturbed eating, depression, and anxiety) was also examined. In the whole sample, a total of 8.5% (4.8% of males and 11.0% of females) had two positive screens (i.e. scored above cut-off for either two of the three measures of psychopathology), whereas 5.4% (2.9% males and 7.1% females) had three positive screens (i.e. scored above the cut-off score for all three measures of psychopathology).

As shown in Table 3, there were no significant differences in metabolic control between patients with versus without DEB, depression, or anxiety. As for age, BMI, and T1D duration, the results were mixed. There were no significant differences between individuals with no psychopathology (zero positive screens) and two positive screens. When comparing individuals with no positive screens versus three positive screens, a statistical significant difference found was in age, demonstrating that individuals with three positive screens were significantly younger than individuals with no positive screens (31.9 years (10.8) versus 43.9 years (15.9), *p* < 01).

**Table 3:** Comparison of participants with and without disturbed eating behavior (below/above the DEPS-R cut-off of ≥20), depression (below/above the HAD depression cut-off score ≥ 11), and anxiety (below/above the HAD anxiety cut-off score ≥ 11)

	DEB -	DEB +	Sig. level	ES	Depr -	Depr +	Sig. level	ES	Anx -	Anx +	Sig. level	ES	No pos screens	2 pos screens	Sig.level	ES	No pos screens	3 pos screens	Sig. level	ES
HbA1c (%)	7.7 (.9)	8.0 (.9)	ns	–	7.7 (.9)	8.0 (.8)	ns	–	7.7 (.9)	7.8 (.8)	ns	–	7.7 (.9)	7.9 (.8)	ns	–	7.7 (.9)	7.9 (.8)	ns	–
BMI	25.5 (3.7)	27.7 (4.9)	.01	0.5	25.8 (3.8)	29.2 (7.1)	ns	–	25.9 (3.8)	26.6 (5.4)	ns	–	25.5 (3.5)	26.9 (5.6)	ns	–	25.5 (3.5)	29.1 (6.5)	ns	–
T1D duration	28.4 (14.8)	24.0 (12.7)	.05	−0.3	27.2 (14.6)	27.5 (12.6)	ns	–	27.8 (14.8)	24.8 (13.1)	ns	–	28.3 (15.2)	31.5 (13.5)	ns	–	28.3 (15.2)	21.3 (11.0)	ns	–
Age (years)	44.1 (15.3)	35.4 (12.4)	.001	−0.6	42.3 (15.4)	39.9 (11.3)	ns	–	43.1 (15.5)	38.3 (13.1)	.05	.3	43.9 (15.9)	43.0 (14.6)	ns	–	43.9 (15.9)	31.9 (10.8)	.01	.9

Data are mean (SD), significance level (*p* < .001, .01, and .05) and effect size (ES) estimation (Cohen’s *d*)

**Fig. 1:**
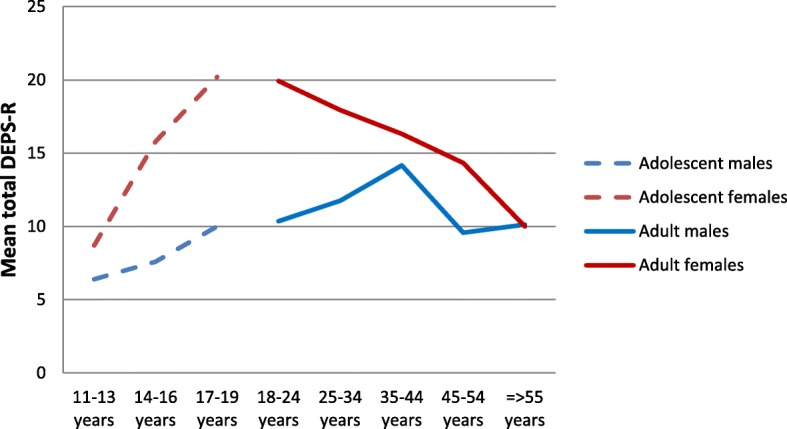
Mean DEPS-R total score according to age group in the current sample of adult males and females with T1D, as depicted by a solid lineNote: Mean DEPS-R total score according to age group in our previous sample of adolescent males and females with T1D [25] is indicated by a dashed line

**Fig. 2:**
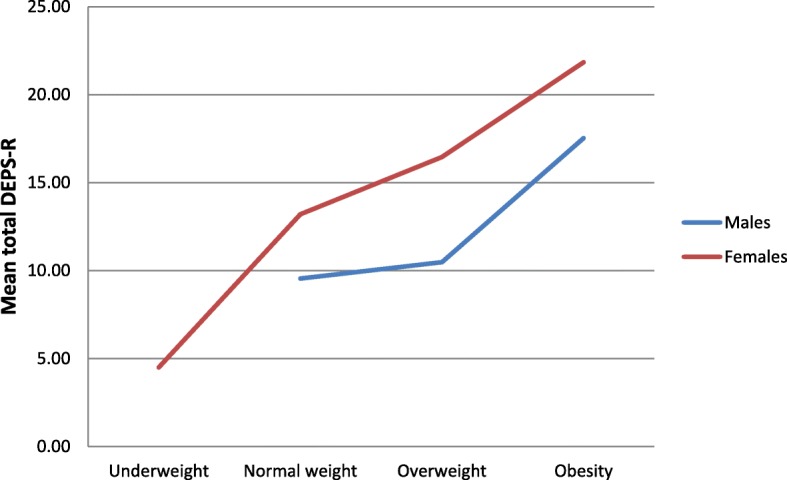
Mean DEPS-R total score in males and females with T1D by BMI categoryNote: BMI was categorized into the following four groups according to the World Health Organization classification scheme (28): underweight (BMI < 18.5), normal weight (BMI ≥ 18.5–24.9), overweight (BMI ≥ 25–29.9), and obese (BMI ≥ 30)

Mean DEPS-R score was 13.83 (9.16) for the total population, 11.18 (7.80) for males, and 15.57 (9.59) for females (*p* < .001, effect size −.50), indicating higher levels of eating disorder psychopathology among females. Table 4 shows the DEPS-R mean scores among males and females according to different categories of age and weight. Figures 1 and 2 further illustrate the distribution of eating disorder psychopathology by the different age and weight groups. The DEPS-R mean score decreased steadily by age among females, whereas the trend for males were more mixed. As for weight, the DEPS-R mean score increased by increasing weight category for both males and females. Mean score for the HADS depression subscale in the total sample, males, and females was 3.75 (3.61), 3.53 (3.35), and 3.90 (3.77), respectively, with no significant difference between males and females. For anxiety, the mean scores for the whole sample was 6.39 (4.27), and 5.12 (3.67) and 7.26 (4.44) for males and females, respectively.

**Table 4:** DEPS-R mean scores in males and females with T1D according to different categories of age and weight

	All	Males	Females	Sig. level
Age				
18–24 years	16.39 (10.4)	10.36 (8.8)	19.92 (9.7)	.01
25–34 years	16.11 (10.5)	11.75 (6.7)	17.95 (11.4)	.05
35–44 years	15.46 (9.9)	14.17 (10.3)	16.32 (9.7)	ns
45–54 years	12.48 (7.1)	9.6 (5.5)	14.33 (7.5)	.05
55–64 years	10.06 (6.9)	10.71 (8.5)	9.6 (5.5)	ns
≥ 65 years	10.00 (6.0)	9.7 (5.2)	10.42 (7.3)	ns
BMI				
Underweight	4.50 (5.0)	–	4.50 (5.0)	–
Normal weight	11.89 (8.6)	9.55 (7.3)	13.20 (9.0)	.05
Overweight	13.89 (8.3)	10.48 (7.1)	16.46 (8.3)	.001
Obese	19.94 (10.5)	17.53 (8.6)	21.84 (11.6)	ns

data are means (standard deviations)

### Associations

Table 5 demonstrates associations between eating disorder psychopathology, depression, anxiety, age, BMI, and HbA1c. Significant associations were found between symptoms of eating disorder psychopathology, depression, and anxiety, with correlation coefficients ranging from .39 (*p* < .001) to .61 (*p* < .001) among males, and .47 (*p* < .001) to .68 (*p* < .001) among females. Furthermore, HbA1c was significantly associated with the DEPS-R total score among females (.27, *p* < .01), but not among males. However, HbA1c was not significantly correlated with the subscale scores of depression and anxiety in neither males nor females. BMI was significantly and positively correlated with eating the DEPS-R total score (.33, *p* < .001) and depression (.30, *p* < .001) among females, but not with anxiety. Among males, BMI was only significantly associated with the DEPS-R total score (.35, *p* < .001), but not with scores of depression or anxiety. Finally, age was significantly and negatively associated with both the DEPS-R and HADS anxiety total scores among females (−.32, *p* < .001 and − .24, *p* < .01, respectively), with lower levels of anxiety and eating disorder psychopathology with higher age (age was not significantly associated with depression). Age was not significantly associated with any of the psychopathology scores among males.

**Table 5:** Associations between psychopathology (disturbed eating, depression, and anxiety), age, BMI, and HbA1c, in adult males (left diagonal) and females (bold, right diagonal) with T1D

Males	Females						
		DEPS-R total	HAD depression	HAD anxiety	Age	BMI	HbA1c
	DEPS-R total	–	.47***	.48***	−.32***	.33***	.27**
	HAD depression	.39***	–	.68***	**ns**	.30***	**ns**
	HAD anxiety	.50***	.61***	–	−.24**	**ns**	**ns**
	Age	ns	ns	ns	–	**ns**	**ns**
	BMI	.35***	ns	ns	ns	–	.19*
	HbA1c	ns	ns	ns	−.24*	ns	–

data are Pearson correlation coefficients, and significance levels are indicated by *s (*p* < .001***; *p* < .01**; *p* < .05*). *Ns* not significant associations

entries in the right diagonale are marked in bold to indicate that they refer to the females of our sample

## Discussion

This study reported the prevalence of DEB among adult males (13.3%) and females (24.8%) with T1D. Level of eating disorder psychopathology decreased with increasing age among females. Furthermore, symptoms of depression and anxiety were reported, with one fourth of females scoring above cut-off for anxiety. Females had generally higher scores on measures of psychopathology in terms of DEB, depression, and anxiety, than males.

### Prevalence of DEB

The observed gender difference in prevalence of DEB supports previous studies of eating disorder psychopathology, both in diabetes [34, 35] and non-diabetes [36] samples. Furthermore, the prevalence increased by increasing weight, which is comparable to pediatric T1D samples [25, 37–39] as well as the ED literature in general [40]. However, it should be noted that BMI generally increases with age, and the current sample is relatively old compared to many studies of comorbid DEB and T1D. In line with previous research [40], the prevalence of DEB was highest among the young adult females, with decreasing eating disorder psychopathology with older age. This is the opposite trend to what has been reported in child and adolescent samples, where rates of disturbed eating have been found to increase by increasing age [39]. This most likely reflects the peak age of eating disorder onset during late adolescence and early adulthood among females [36]. This peak in DEB during late adolescence and early adulthood is also evident in Fig. 1, illustrating levels of DEB according to different age groups in our previous adolescent sample [39] and the current adult sample. Older adolescents and young adults with T1D is generally found to be in a vulnerable phase as they are in the process of transferring from pediatric to adult health care, indicating lower levels of care and support. Additionally, individuals at this point in life typically move away from their home for the first time [41–43].

### Prevalence symptoms of anxiety

This study found that 8.1% of males and 26.4% of females scored above the cut-off score for anxiety at 11 or above (HADS-A). These rates are comparable to those reported by Lloyd et al. [44] among their mixed sample of patients with both T1D and type 2 diabetes (T2D). Using the same cut-off as the current study, a total of 25% scored above cut-off for moderate to severe symptoms of anxiety. Females tended to report more moderate-severe anxiety than males. There were no significant differences between types of diabetes. Another study used the 8 or above cut-off score on the HADS-A, and found that 22.4% of the males and 37.2% of the females had mild symptoms of anxiety. Furthermore, a systematic review of diabetes and anxiety [14] reported prevalence rates ranging from 15 to 27.5% when using the cut-off score 8 on the HADS-A. As can be expected with the use of a lower cut-off, these rates are somewhat higher than those reported in our study, and in the study by Lloyd et al. [44].

The current study did not include a control group to compare rates of anxiety in this T1D population to those of non-diabetes controls. However, a large Norwegian population study (the HUNT 2 study) has been conducted, reporting levels of anxiety in the general population, aged 20–89 years (*N* = 60.869). Bjelland et al. [45] reported that 9.6% scored above the cut-off score of 8 for anxiety. When using the same cut-off score in our sample of both males and females with T1D, a prevalence of 35.4% was reported for anxiety. This is much higher than the prevalence reported in the HUNT study, suggesting that levels of anxiety are higher among patients with T1D than non-diabetes controls.

As the HADS is a generic measure, it should be noted that reported symptoms of anxiety in the current study may be driven by diabetes-specific distress. Previously reported diabetes-specific features of anxiety include fear of complications, fear of hypoglycemia, and invasive self-care behaviors such as fear of injections, self-monitoring of blood glucose, and insertion of subcutaneous insulin infusion devices such as an insulin pump [10]. Such illness-specific aspects may contribute to higher rates of anxiety among patients with diabetes.

### Prevalence symptoms of depression

A total of 11.8% of the males and 7.8% of the females scored above the cut-off score for depression (HADS-D) in the present study, indicating moderate to severe symptoms of depression, or “caseness”. Lloyd et al. [44], using the same cut-off, found that 8% of the patients with diabetes scored above the cut-off for depression, thus in line with our study. Knychala et al. [46] used the cut-off score of 8, and reported prevalence rates of 4.7% among males and 17.6% of females with diabetes. Given the lower cut-off, the higher prevalence rates compared to the previous two studies, can be expected. The meta-analysis by Anderson et al. [13], however, reported higher prevalence rates, with rates for elevated depressive symptoms being 21.3% for adults with T1D. Rates of depressive disorders, as assessed by a diagnostic interview, ranged from 8 to 15% in adults with T1D and T2D (no studies investigated rates in samples with T1D only). A systematic review by Roy et al. [47] reported a three-fold increase in prevalence of depression among individuals with T1D compared to those without, with prevalence rates ranging from 5.8 to 43.3% (range among individuals without diabetes was 2.7% to 11.4%).

As for comparison with Norwegian control data, the prevalence of depression when using the cut-off score of 8 was 4.9% in the HUNT 2 study [45]. When using the same cut-off in the current study, we found a prevalence of depression at 13.8%, indicating that levels of depression are higher among individuals with T1D than individuals without.

The varying prevalence rates across studies underlines the importance of considering methodological aspects when interpreting reported prevalence rates. It is important to note that reported prevalence rates are highly dependent on the adopted cut-off score, and there are variations in the literature as to which cut-off score is utilized. We chose to adopt the more conservative cut-off 11 to minimize the risk of over pathologizing. The adopted cut-off score is important to take into account when interpreting prevalence rates across studies. Finally, it should be noted that screening measures cannot be used to establish a diagnosis as defined by diagnostic manuals. This can only be done with clinical diagnostic interviews. Screening measures of psychopathology may yield inaccurate estimates, but they are a simple and quick method. Therefore, it is recommended to validate positive screens with an interview.

### Above versus below cut-off on psychopathology

There was no significant difference in HbA1c among patients scoring above versus below the DEPS-R or the HADS depression or anxiety cut-off scores. Eating disorder psychopathology (mean DEPS-R total score) was, however, significantly and positively associated with metabolic control in females, but this was not the case for depression or anxiety. It is not clear why there was no significant difference in HbA1c between individuals scoring above versus below cut-off on the DEPS-R when the association between the DEPS-R total score and HbA1c is significant. One potential reason is that the analysis is less sensitive when the data are dichotomous rather than dimensional. It should also be mentioned that despite the presence of DEB, depression, and anxiety, the HbA1c in the current sample is relatively good. Further, the percentage of patients using an insulin pump is relatively high. These factors might indicate that the patients receive good T1D care within a multidisciplinary team, including psychological health care personnel. The lack of associations between depression/anxiety and metabolic control is in contrast to several other studies [15–18], although not all [23, 48]. The discrepancies in this regard across studies may be explained by various factors. For example, it has been argued that it has been suggested that distinct profiles of depression exist, which may impact outcome differently [49]. Also, it has been shown that diabetes-specific emotional distress, not depression, is associated with metabolic control [23]. These two concepts are commonly being used interchangeably, despite the fact that they are not overlapping constructs [49, 50]. An underlying construct of diabetes-specific emotional distress be considered as a core structure to link diabetes-related distress, subclinical depression, elevated depression symptoms, and major depressive disorder [50]. Targeting illness-specific cognitions may be more productive than treatment of general dysphoria in T1D [22].

Although studies have found significant relationships between the HADS scales and metabolic control [44], the lack of significant relationship between anxiety/depression and metabolic control in our study may also be explained by the use of this measure, which was specifically designed for patients within a hospital setting. To avoid false positives, i.e. avoid symptoms of somatic illness to be falsely interpreted as psychopathology, somatic symptoms of depression and anxiety are omitted in the HADS. As some of the symptoms of anxiety and depression in diagnostic manuals are indeed of somatic character, this may have influenced the expected association between these variables and metabolic control in the current study. In fact, Bot et al. [16] reported that somatic symptoms of depression were some of the symptoms of depression most strongly associated with metabolic control, which may explain the lack of significant association between depression and metabolic control in this study. This assumption may be supported by another Norwegian study, which also used the HADS, and did not find a significant association between depression and HbA1c [23]. However, they did find a significant association between diabetes-related distress and metabolic control, supporting the suggested distinction between depression and diabetes-related distress as discussed above. Taken together, this suggests that various related correlates may play a role in explaining the relationship, or lack thereof, between depression/anxiety and metabolic control, and that illness-specific distress could be taken into account.

Participants scoring above cut-off for DEB and anxiety were significantly younger than participants scoring below cut-off. Similarly, patients with three positive screens (i.e. scored above cut-off for DEB, depression, and anxiety) were significantly younger than patients scoring below cut-off on all three measures of psychopathology. Decreasing eating disorder psychopathology by increasing age among adults is in line with previous eating disorder literature among individuals without T1D [40]. Similar to the negative correlation between eating disorder psychopathology and age, patients scoring above cut-off for anxiety were significantly younger than those scoring below. Previous literature has shown evidence of decreased susceptibility to both depression and anxiety with increasing age [10, 47]. This is only partially in conjunction to the current study, since the negative correlation between depression and age was not statistically significant.

The significant associations between age and measures of psychopathology are also evident in the correlation analyses (Table 4). Furthermore, and as expected, symptoms of DEB, depression, and anxiety were significantly associated with each other, with medium to large correlations. Finally, this study specifically aimed to inspect the relationships between eating disorder psychopathology with age and weight. As illustrated by Fig. 1, DEPS-R mean scores decreased by increasing age among females. This is in line with findings from the eating disorder literature among adults [40]. In contrast, eating disorder psychopathology has been found to increase by increasing age among adolescent samples [39]. These different age patterns most likely reflect the peak onset of eating disorders during the late adolescence [51]. Further, as demonstrated in Fig. 2, eating disorder psychopathology in the current study increased by increasing weight. This is consistent to previous research among adults in the eating disorder literature [40], as well as with adolescents with T1D [39].

The inclusion of males and older females represents a strength of this study as the majority of existing literature is focused on adolescent and young adult females only. Also, the use of the diabetes-specific measure DEPS-R is a strength and in line with current recommendations [5] to yield more accurate estimates of prevalence rates. However, the cross-sectional design is a weakness as we cannot infer causality. More studies are needed to elaborate on the directions of the relationships between diabetes and psychopathology. Also, data was collected from only one diabetes clinic, and we can therefore not be sure whether the results of this study are representative for the entire adult T1D population. Furthermore, the glycemic control was not measured at the same time as the psychological assessment. Finally, the data gathered in this study was self-reports only, and we can therefore not establish formal eating disorder diagnoses as defined by diagnostic manuals such as the DSM-5.

## Conclusion

In conclusion, the present study points to the need for awareness of psychological comorbidity among patients with T1D. One fourth of females suffer from symptoms of anxiety and DEB, which is likely to negatively impact quality of life, a prioritized aim for diabetes treatment, regardless of metabolic control. Screening can be recommended to secure early detection and subsequent intervention, in particular among young adult females. Finally, the effects of age should be recognized, as older adolescent [39] and young adult females with T1D appear to be in particular risk of developing eating disorder psychopathology and symptoms of anxiety. This coincides with age of transitioning from pediatric to adult health care, which is generally described to be a vulnerable phase as discussed above. Such aspects point to the need for clinicians to be particularly aware of this age group in terms of eating disturbances.

## Abbreviations

BMI: Body mass index
CDC: The center for disease control and prevention
DEB: Disturbed eating behavior
DEPS-R: The diabetes eating problem survey – revised
DSM: The diagnostic and statistical manual of mental disorders
HADS: The hospital anxiety and depression scale
HADS-A: The hospital anxiety and depression scale; anxiety subscale
HADS-D: The hospital anxiety and depression scale; depression subscale
HbA1c: Hemoglobin A1c
HUNT: The Nord-Trøndelag health study
NDC: The Norwegian diabetic center
NOKLUS: The Norwegian quality improvement of laboratory examination
SPSS: The statistical package for the social sciences
T1D: Type 1 diabetes
T2D: Type 2 diabetes
